# A dyadic approach of assessing the therapeutic alliance in youth mental health and addiction treatment

**DOI:** 10.1007/s00787-025-02784-9

**Published:** 2025-06-13

**Authors:** Patty van Benthem, Renske Spijkerman, Peter Blanken, Robert R. J. M. Vermeiren, Vincent M. Hendriks

**Affiliations:** 1 Department of Child and Adolescent Psychiatry, LUMC Curium, Leiden University Medical Center, Leiden, The Netherlands; 2 Parnassia Addiction Research Centre (PARC), Brijder Addiction Care, The Hague, The Netherlands

**Keywords:** Therapeutic alliance, One-with-many design, Social relations model, Treatment outcome, Substance use disorder, Youth mental health

## Abstract

When studying therapeutic alliance, researchers typically use data with a ‘one-with-many’ (OWM) nested structure in which each therapist (‘one’) treats several clients (‘many’). The OWM structure offers the opportunity to assess the interdependence in dyadic therapeutic alliance data by disentangling three components: (1) the client’s tendency to generally rate the alliance with his therapists (‘client perceiver’ component); (2) the client’s rating of the unique alliance with his therapist (‘client relationship’ component); and (3) the therapist’s characteristics that elicit a consistent alliance rating in his clients (‘therapist partner’ component). Similarly, three such components can be distinguished for the therapists’ alliance ratings. We used the reciprocal OWM design to distinguish these alliance components, pertaining to the first therapeutic session of two hundred and three youths aged 13 to 23 years who entered a youth mental health and youth addiction treatment facility. We found that most of the alliance variance pertained to the relationship component (client-rated alliance: 99.2%; therapist-rated alliance: 56.0%), with virtually no evidence of therapist’s characteristics that elicited a consistent (high or low) alliance rating in his clients. Clients who reported a uniquely strong alliance with their therapist were more likely to have a favorable 4-months treatment outcome (Odds ratio 1.10; p < 0.001). None of the other (client/therapist) alliance components were related to treatment outcome, nor did we find evidence of dyadic reciprocity, which would occur when a therapist would report a particularly strong alliance with the client if that client reported a particularly strong alliance with the therapist, and vice versa.

**Supplementary Information:**

The online version contains supplementary material available at 10.1007/s00787-025-02784-9.

## Introduction

Research among patients in mental health treatment has shown a consistent, positive association between the quality of the therapeutic alliance and treatment outcome across types of treatment and alliance perspectives (e.g., patient or therapist) in both adult [1, 2] and youth studies [3–5]. The most recent and comprehensive multi-level meta-analysis of alliance-outcome associations in child and adolescent psychotherapy reported a mean effect size of *r* = 0.17 (*p* < 0.001, 62 studies) [5] which was smaller than the effect size in the largest meta-analysis on adult psychotherapy by Flückiger et al. [1] (*r* = 0.28, *p* < 0.0001, 295 studies). One explanation for the smaller alliance-outcome associations in child and adolescent therapy may stem from different conceptualizations of the therapeutic alliance between youth and adults [5]. Due to developmental and contextual differences, youth may prioritize different aspects of the alliance compared with adults, and building a constructive therapeutic alliance with adolescent clients may require a somewhat different approach [6, 7].

In youth, studies that included both the alliance perspective of the youth and the therapist demonstrated generally moderate to low [8–15] or nonsignificant agreement [12–17] between these two perspectives. Most previous studies that investigated the therapeutic alliance from more than one perspective were in search of the “best” perspective - i.e., which perspective showed the strongest association with treatment outcome. However, there appears to be no “best” perspective: both perspectives on the alliance appear to be predictive of outcome, with effect sizes depending on study characteristics and methodological approach [3, 5].

Perhaps more importantly, the focus on just one perspective tends to ignore the dyadic nature of the therapeutic relationship. The relationship between client and therapist in co-creating the alliance is emphasized in Bordin’s [18] conceptualization of the therapeutic alliance as a partnership. Accordingly, alliance ratings do not refer to a single person or a single perspective but rather to the interaction between the two persons in their relationship [19, 20]. Hence, evaluations of the therapeutic alliance and its association with treatment outcome need to reflect both the client-therapist relationship as well as the client and therapist as individuals.

The social relations model (SRM) [21] provides a conceptual and statistical approach for understanding and analyzing perceptions and actions that occur between pairs of individuals, which can also be applied to the assessment of therapeutic alliance in client-therapist dyads. A modification of the SRM– the ‘one-with-many’ (OWM) model [22]– follows the nested structure which is common in virtually all individual psychological treatments, in which each therapist (‘one’) treats multiple clients (‘many’), but each client is not treated by multiple therapists. Concerning therapeutic alliance in a reciprocal OWM model, in which both the client and therapist rate the alliance, this implies that multiple clients rate the alliance with their therapist, but each client is only rated by one therapist.

**Fig. 1:**
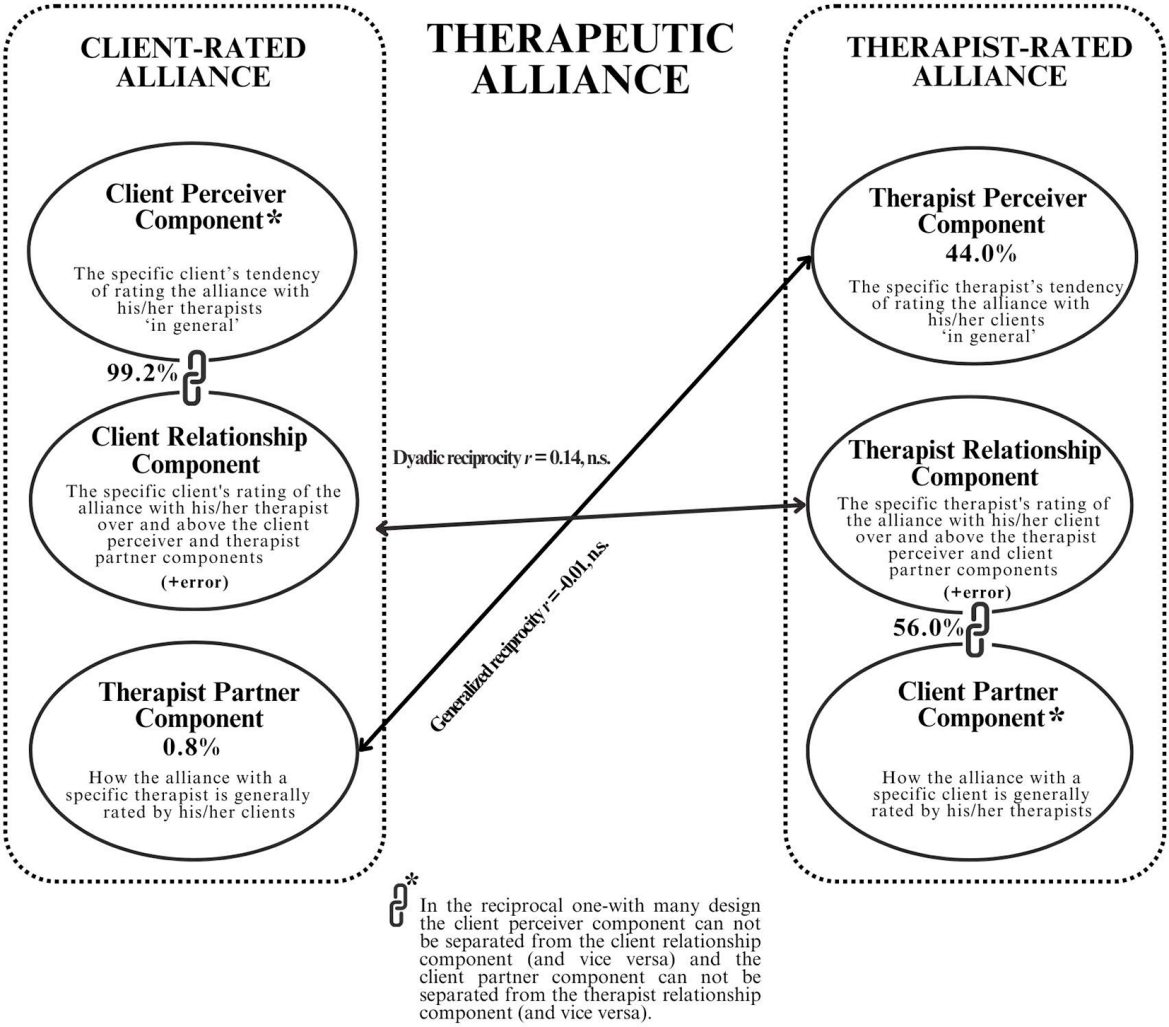
The Variance Components of the Client- and Therapist-Rated Therapeutic Alliance in the Social Relations Model

Components of variance in the one-with-many model.

When considering the therapeutic alliance in a reciprocal OWM design, three client-related and three therapist-related alliance components are involved (please see Fig. 1). Regarding the client-rated alliance depicted at the left side of Fig. 1, if a client reports a strong alliance with his/her therapist, this in part includes (1) the specific client’s tendency to rate the alliance with therapists ‘in general’ as strong, i.e., regardless of who his/her actual therapist was (the client perceiver component), (2) the specific therapist’s traits and characteristics which elicit a consistent strong alliance rating in most of his/her clients (the therapist partner component), and (3) the specific client’s unique relationship with his/her specific therapist, in which the client rates the alliance as stronger than he/she would have with most other therapists, and stronger than the ratings given by most of the therapist’s other clients, i.e., over and above the client perceiver and therapist partner components (the client relationship component).

Similarly, concerning the therapist-rated alliance depicted at the right side of Fig. 1, if a therapist reports a strong alliance with his/her client, this in part contains (4) the specific therapist’s tendency to rate the alliance with clients ‘in general’ as strong, regardless of who his/her actual client was (the therapist perceiver component), (5) the specific client’s traits and characteristics which elicit a consistent high alliance rating in most therapists (the client partner component), and (6) the specific therapist’s unique relationship with his/her specific client, in which the therapist rates the alliance with the client as stronger than for his/her typical client, and stronger than the ratings most other therapists would have given had they been treating this client, i.e., over and above the therapist perceiver and client partner components (the therapist relationship component).

The reciprocal OWM design presents two major advantages over commonly employed analytical approaches in alliance research. First, the OWM design takes the interdependence in alliance data into account by distinguishing different alliance components. Second, the interdependence in the data can be used to estimate generalized reciprocity– are therapists who report strong alliances with their clients also perceived to form strong alliances by their clients (i.e., the correlation between the therapist-receiver and therapist partner component; see Fig. 1), and dyadic reciprocity– if a client reports a unique strong alliance with the therapist, does the therapist also report a unique strong alliance with that client (the correlation between the client relationship and therapist relationship component). However, compared with the SRM ‘round-robin’ structure in which each individual rates and is rated by every other individual, the reciprocal OWM-design has a limitation in differentiating all alliance components. Because in the reciprocal OWM design each client provides alliance data pertaining to his or her therapist only, and not multiple therapists, the client perceiver component cannot be separated from the client relationship component (and vice versa), because we do not know whether the client would have provided different alliance ratings had he/she been treated by a different therapist. Moreover, because only one therapist provides alliance data regarding one client, the client partner component cannot be differentiated from the therapist relationship component (and vice versa), because we do not know if different therapists who had treated the same client would have provided similar alliance ratings. Therefore, in the reciprocal OWM design four instead of six sources of variance can be distinguished: the therapist-partner and client relationship (+ client perceiver) components in client-rated alliance and the therapist-perceiver and therapist relationship (+ client partner) components in therapist-rated alliance.

Despite the advantages of the reciprocal OWM design this design is rarely used in alliance studies. Only three studies on therapeutic alliance to date - two among adults [23, 24] and one among youth [25] - have used this design to partition alliance variance components and investigate their association with treatment outcome. These studies showed consistent findings regarding variance partitioning, dyadic reciprocity, and a positive relation between the combined client relationship + client perceiver component and treatment outcome. However, therapist-related associations with treatment outcome showed equivocal results.

The present study is part of the Professional Alliance with Clients in Treatment (PACT) initiative in which we earlier investigated the value of incorporating first-session therapeutic alliance ratings from both clients and therapists for predicting 4-months treatment outcome in a youth mental health and youth addiction treatment setting, which showed that youth with a strong alliance according to both perspectives had an eightfold odds of favorable treatment outcome compared with youth with a weak alliance according to both perspectives. In a second study among these youths, we conducted a controlled trial in which we compared a client feedback intervention as an add-on to treatment as usual (TAU) versus TAU-only, which indicated that the client feedback intervention did not show better 4-months treatment outcomes or better alliances than TAU [26]. In the present study in the same youth sample, we used the reciprocal OWM model to investigate the composition of the therapeutic alliance at the first treatment session, with regard to (1) the amount of variance accounted for by the various therapeutic alliance components– with a specific focus on the therapist perceiver and therapist partner component, (2) the presence or absence of generalyzed and/or dyadic reciprocity, and (3) the association between the client-rated and therapist-rated alliance components with treatment outcome.

## Method

### Participants

The present study among youth in mental health and addiction treatment included 203 clients, aged 13 to 23 years, and 61 therapists with first-session therapeutic alliance ratings completed by both client and therapist. Clients were on average 18.2 years old, half of the clients were female (50.2%), and most had a Dutch-western cultural background (74.9%). Most clients in youth addiction treatment had a primary cannabis use disorder (50.0%), followed by alcohol use disorder (18.8%) and hard drug use disorder (12.5%). Most youth in mental health treatment had a primary anxiety disorder (28.0%), mood disorder (27.1%), or behavioral disorder (17.8%). Therapists were on average 38.2 years old, and most of the therapists were female (79.0%) and had a Dutch cultural background (90.0%). The therapists’ caseloads ranged from 14 therapists who treated 1 client; 16 therapists 2 clients; 10 therapists 3 clients; 8 therapists 4 clients, up to 1 therapist treating 16 clients (mean = 3.3 clients per therapist; median = 4).

### Assessments

Data were collected by trained research assistants who were independent from treatment staff. Immediately following the first treatment session, patient- and therapist-rated therapeutic alliance was assessed, using the client and therapist version of the 12-item Working Alliance Inventory– Short version (WAI-S) translated into Dutch (WAV-12) [27]. Clients and therapists were required to rate each item on a 5-point Likert scale, ranging from ‘never’ (1) to ‘always’ (5) with higher scores indicating higher quality of the alliance. Internal consistency of the WAI-S at the first treatment session in our study was good, with Cronbach’s alpha = 0.88 (youth version) and alpha = 0,89 (therapist version) [28]. Youth self-reported mental health problems at baseline and 4 months post-baseline were assessed with the Strengths and Difficulties Questionnaire Self Report (SDQ-SR) [29, 30], a commonly applied instrument to screen and monitor psychosocial problems in children and adolescents [31]. The questionnaire contains 20 items focusing on difficulties that can be rated on a 3-point Likert scale ranging from ‘not true’ (0) to ‘certainly true’ (2). We used the SDQ-SR total difficulties score (range 0–40), with higher scores indicating more problems. The SDQ-SR has been validated in adolescents, aged 11 to 17 years, in different countries, with satisfactory psychometric properties in both community and clinical samples of Dutch adolescents [32, 33]. A slightly adapted version of the SDQ-SR has shown similar psychometric properties in young adults [34]. Cronbach’s alpha of the SDQ total scores in our study at baseline and 4 months follow-up were acceptable, with alpha = 0.74 and 0.69, respectively. Youth self-reported addiction problems were measured using the substance use section of the Measurements in the Addictions for Triage and Evaluation, Youth version (MATE-Y) [35] to assess the number of substance use days of the clients’ primary addiction in the past month.

### Primary outcome measure

We used the SDQ and the MATE-Y to obtain a dichotomous outcome measure reflecting a favorable versus unfavorable treatment outcome status at 4-month follow-up for clients in youth mental health treatment and youth addiction treatment, respectively. Youth in mental health treatment were considered to have a favorable outcome if their SDQ total score at month-4 was lower than the clinical threshold of 12.5. Lacking formal Dutch SDQ cut-off scores [36], we followed the procedures of Jacobson and Truax [37] and De Beurs et al. [38], to determine the cut-off value of 12.5 as the average of the mean SDQ total score in a Dutch general youth population sample (M = 9.7, SD = 4.7 [39], and the mean baseline SDQ total score in our clinical population (M = 15.3, SD = 5.4). Youths in addiction treatment were considered to have a favorable outcome if they had used their primary substance on less than five days in the 30 days preceding the 4-month follow-up, as recommended in the guidelines for routine outcome monitoring (ROM) in Dutch addiction care (Blanken et al., 2011, Note from Dutch Expertgroup ROM-Addiction care).

### Data-analysis

We conducted the reciprocal OWM analysis, using multilevel modeling analysis (MLM) and the datafile structure and SPSS code as proposed by Kenny, Kashy, and Cook [40] SPSS version 27 (IBM Corp., Armonk, N.Y., USA). We structured a data set with six variables. The first two variables were client ID (YTHID; numbered from 1 to 203) and therapist ID (THERID; numbered from 1 to 61. The second two variables were the client’s alliance score (YTHWAI) and the therapist’s alliance score for that client (THERWAI). The last two variables were the mean client’s alliance scores across all clients of a particular therapist (YTHWAI_mean) and the mean therapist’ alliance score across all their clients (THERWAI_mean). To examine the proportions of variance in the therapist partner; client relationship (+ client perceiver); therapist perceiver; and therapist relationship (+ client partner) components, parameter estimations were made using restricted maximum likelihood (REML) with an unstructured correlation’s structure. Wald-z tests were used to determine the significance of the different variance components. Regarding reciprocity, we computed the generalized reciprocity by calculating the correlation between the therapist perceiver and the therapist partner variances and the dyadic reciprocity by calculating the correlation between the client relationship and the therapist relationship variances. Finally, we examined associations between the four alliance components mentioned above and our dichotomous outcome variable (favorable vs. unfavorable treatment outcome status). Missing 4-month outcome assessments (*n* = 33) were estimated by the treating therapist.

## Results

### Variance partitioning

The variance partitioning for the client-rated and therapist-rated WAI-S at the first treatment session is illustrated in Fig. 1. In the client-rated alliance, the therapist partner component accounted for a small (0.8%) and non-significant amount of the variance (Wald *Z* = 0.136, *p* = 0.90), which indicates that among clients of a specific therapist there was not much consensus about the quality of the therapeutic alliance. Thus, from the client’s perspective, there was no evidence that some therapists overall had a better (or worse) alliance with their clients than other therapists. Concerning the therapist-rated WAI-S, the therapist perceiver component (Wald *Z* = 3.60, *p* < 0.001) accounted for 44% of the variance, indicating that almost half of the variance could be attributed to the specific therapist’s tendency to rate the alliance with clients in general. Most of the variance in both clients’ and therapists’ alliance ratings (99.2% and 56%, respectively) could be attributed to the (undifferentiated) client relationship and client perceiver component; Wald *Z* = 8.57, *p* < 0.001) and the (undifferentiated) therapist relationship and client partner component; Wald *Z* = 8.54, *p* < 0.001).

### Reciprocity

The generalized reciprocity correlation between the therapist perceiver and the therapist partner component was non-significant (*r* = -0.01, *p* = 0.86), indicating that therapists who generally reported strong alliances with their clients were not generally rated by their clients to form strong alliances (and vice versa). The dyadic reciprocity correlation between the undifferentiated client relationship plus client perceiver component and the undifferentiated therapist relationship plus client partner component was not significant either (*r* = 0.14, *p* = 0.06): when a particular client reported a uniquely strong alliance with his/her therapist– stronger than reported by other clients of this therapist– the therapist did not report a uniquely strong alliance with this particular client– stronger than reported with other clients (and vice versa).

### Alliance and outcome

The model parameter estimates of the analysis regarding the association between alliance and treatment outcome are specified in Table 1. Regarding the client-rated alliance we found that the therapist partner component was not associated with treatment outcome, *b* = 0.02 (*p* = 0.736; OR 0.98), indicating that therapists with whom multiple clients generally reported a strong alliance were not more likely to have clients with a favorable treatment outcome. The client relationship component (+ client perceiver component) was significantly associated with outcome, *b* = 0.09 (*p* < 0.001; OR 1.10). Clients who reported a uniquely strong alliance with their therapist were more likely to have a favorable treatment outcome. Regarding the therapist-rated alliance we found that the therapist perceiver component was not significantly associated with treatment outcome, *b* = 0.08 (*p* = 0.121; OR 1.09). Hence, therapists who reported strong alliances across their clients ‘in general’ were not more likely to have clients with a favorable treatment outcome. In addition, there was no association between the therapist relationship component and outcome, *b* = 0.03 (*p* = 0.491; OR 1.03), indicating that therapists who reported a uniquely strong alliance with their clients were not more likely to have clients with a favorable treatment outcome.

**Table 1:** Parameter estimates for the association between alliance and treatment outcome

Parameter	b	OR	95%-CI of OR	*p*
Intercept client WAI-S ratings	-9.19	0.00	3.11^E − 7^- 0.03	0.002
Therapist Partner	0.02	0.98	0.89 − 1.09	0.736
Client Relationship (+ client perceiver)	0.09	1.10	1.04 − 1.16	0.001
Therapist Perceiver	0.08	1.09	0.98 − 1.21	0.121
Therapist Relationship (+ client partner)	0.03	1.03	0.95 − 1.11	0.491

## Discussion

In this study we aimed to disentangle the various alliance components in clients’ and therapists’ reciprocal ratings of first session therapeutic alliance and their associations with treatment outcome in youth mental health and addiction care using a one-with-many (OWM) design. Our study has four main findings. First, the variance in clients’ alliance ratings was almost fully (99.2%) explained by the combined client relationship and client perceiver component, with less than 1% of variance remaining for the therapist partner component, which suggests there was hardly any consensus about the quality of the therapeutic alliance among clients treated by the same therapist. Second, most variance in therapists’ alliance ratings was accounted for by the combined therapist relationship and client partner component (56%), with the remaining 44% attributable to the therapist’s tendency to report similar alliances across his/her clients (therapist perceiver component). Third, we found no indication of generalized or dyadic reciprocity. Hence, therapists who considered themselves as having strong alliances with their clients were not necessarily perceived to have strong alliances by their clients, and if a therapist reported a particularly strong alliance with a specific client, that client was not more likely to report an especially strong alliance with the therapist. Fourth, clients who reported a uniquely strong alliance with their therapist (i.e., combined client relationship and client perceiver component) were more likely to have a favorable treatment outcome. None of the other client- or therapist-related alliance components were associated with treatment outcome.

Overall, our findings regarding variance partitioning are remarkably similar to the results of previous studies using this type of OWM analysis [23–25, 41]. In each study (see supplementary section, Table S1), the highest amount of variance in alliance ratings could be attributed to the combined client relationship + client perceiver component (> 90% of the variance), followed by the combined therapist relationship + client partner component (55 to 70%), followed by the therapist perceiver component (30 to 45%), and lastly the therapist partner component (< 9%). In addition, all three alliance studies found a positive association between the client relationship (+ client perceiver) component and treatment outcome. Hence, these findings appear to be robust across studies with quite different client sample sizes (*n* = 112 to 439), mean client-therapist ratios (3.3 to 28.4 clients per therapist), age groups (adolescents and adults), timing of the alliance assessment (end of first session vs. end of third or prior to fourth session) and even the main topic under investigation– therapeutic alliance vs. perceived teamness in patient-physician communication [41]. Divergent from the earlier alliance studies, we found no association of the therapist partner component [23] or therapist relationship component [24] with treatment outcome, and no evidence of generalized or dyadic reciprocity. The latter finding may be due to our early assessment of therapeutic alliance immediately following the first treatment session, at a time in which strong reciprocal processes involved in building and strengthening alliance are not yet likely to occur.

The clinical implications of our study findings are not immediately obvious, partly because our negative findings regarding reciprocity and therapist-related associations with treatment outcome contradict some of the findings of the earlier OWM studies mentioned. Nevertheless, all studies appear consistent in suggesting that the strength of the therapeutic alliance is mostly determined by the unique client-therapist relation, and that, from the client’s perspective, therapists’ general skills or characteristics likely do not play a major role in establishing a strong alliance and/or a favorable treatment outcome. These findings suggest that it may be more fruitful to investigate in experimental studies focusing on core elements in the unique client-therapist dyadic interaction and communication patterns– e.g., the ability to navigate through difficult interactions and the sensitivity to adjust to specific client needs– than to invest in research aimed at identifying or strengthening therapists’ general skills [23–25, 41]. Lastly, all reviewed OWM alliance studies concur on finding a considerable therapist perceiver effect, with some therapists consistently providing stronger alliance ratings to their clients than other therapists. This finding suggests the presence of a considerable tendency, or ‘response set’, of therapists to report general alliance ratings with their clients, as opposed to ratings specifically focusing on each unique client. Hence, it is probably wise to provide training to raise therapists’ awareness of the existence of such a response set, to limit its effect in clinical practice.

Our study has several limitations. These include, first and most prominent, the inability to separate the client perceiver and client partner variance from the (client/therapist) relationship variance, due to the fact that each client only has one therapist, which is commonly the case in clinical practice. Although this limitation is inherent to the OWM design, one might argue that a therapist’s tendency to provide his clients with a similar/general alliance rating (therapist perceiver effect; in our study 44%) may not differ from a client’s tendency to rate the alliance with therapists in a similar general way (client perceiver effect; unknown), and that such ‘trait-like’ tendencies [42] may be particularly present during the first treatment session, when very little is known about the other person. If that would be the case, a sizable 44% of the combined client relationship and client perceiver variance could be attributable to the client perceiver component, leaving the remaining proportion of variance (55%) attributable to the unique client relationship. Along the same line of reasoning, the (known and small) therapist partner component may not differ from the (unknown) client partner component, again leaving the majority of the remaining variance attributable to the unique therapist relationship. Hence, if these assumptions are correct, it is quite plausible that most alliance variance is indeed accounted for by the unique client-therapist relationship.

Second, we measured therapeutic alliance as early as possible in the treatment process to minimize possible confound due to early symptom improvement, and to be able to serve as an early warning sign of client/therapist dissatisfaction, possible premature treatment termination and poor treatment outcome. The downside of this early assessment is that it may not be ‘alliance’ per se what is measured but, e.g., likeability, first impressions of motivation, treatment readiness, or other overlapping constructs. Nevertheless, in one of our earlier papers first-session alliance did show a moderately strong association with treatment outcome, with an eight times higher odds of favorable outcome in the subgroup with a strong alliance according to the perspective of both clients and therapists, compared with the odds in the subgroup with a weak alliance from both perspectives [17]. Third, we a-priori defined our primary outcome measure as (un)favorable treatment outcome status at 4 months post-baseline. However, results showed that more than three-quarter of the clients were still in treatment at that time, so patients may have been at different recovery stages at this ‘endpoint’. Nevertheless, many studies in the mental health field indicate that (1) the largest symptom improvement occurs in the early phase of treatment, (2) later treatment phases show relatively little additional symptom improvement, and (3) improvement in the early phase of treatment– or lack thereof– is associated with final treatment outcome [43, 44]. Based on these findings, we believe that our choice of the month-4 endpoint is well defensible. Fourth, whereas eligible study youths were 13 to 22 years old, more than half of youths in the mental health treatment setting were 16 years or younger, as opposed to less than one-fifths of youths in substance use disorder treatment. Because the younger subgroup more likely entered treatment predominantly at the initiative of caregivers and most of the ‘older’ youths likely sought treatment at their own initiative, the role of parents may have been much more relevant for the younger part of the sample than for the older part. However, we have no data in this study to corroborate the parents’ role in their child’s treatment admission, and despite the age difference between both treatment settings, youths’ and therapists’ alliance ratings did not differ between the age groups and treatment settings. Fifth, despite the designation ‘one-with-many’, the 61 therapists in our sample of 203 clients treated only 3.3 clients on average, with a median of 4, which may have limited statistical power to test client-level effects. However, these data are quite similar to the OWM study of Marcus et al. (2009) [23], which included 65 therapists and 227 clients– an average of 3.5 clients per therapist. While Marcus and colleagues found significant dyadic reciprocity and significant associations with treatment outcome, virtually no such effects were found in our study. We therefore do not believe that lack of power to detect client-level or therapist-level effects played a major role in our mostly negative findings. Sixth, we used a dichotomous outcome measure that was merely based on the patients’ 4-months post-baseline outcome status. Using a continuous pre-to-post-baseline improvement measure was not possible because a sizeable subgroup of youths at baseline did not meet the problem-threshold on the SDQ (30.0%) or the MATE-Y (39.7%) and, therefore did not have the opportunity to become a ‘responder’ at 4-month follow-up. While continuous outcomes provide more heterogeneity, statistical power, and the possibility to calculate effect sizes, dichotomous outcomes often have the advantage of inherent clinical clarity and provide the possibility to designate individual patients as ‘responder’ or ‘non-responder’ [45].

## Conclusion

To conclude, the current study provides an in-depth look at the dyadic relationship between youth in mental health and addiction treatment and their therapists, and it provides clues as to which components of the alliance are associated with better treatment outcomes. Our findings suggest that clients who report a uniquely strong alliance with their therapist are more likely to have a favorable treatment outcome, whereas therapists’ alliance rating tendencies and therapists’ general traits and characteristics seem less related to treatment outcome.

## Electronic supplementary material

Below is the link to the electronic supplementary material.

Supplementary Material 1

## Data Availability

The data that support the findings of this study are available on request from the corresponding author, [P.v.B.]. The data are not publicly available due to restrictions to ensure the privacy of minor participants.
