# Supplementary material for: A dyadic approach of assessing the therapeutic alliance in youth mental health and addiction treatment
Source: Eur Child Adolesc Psychiatry. 2025 Jun 13;34(12):3811–9. doi: 10.1007/s00787-025-02784-9 (PMC12743090; doi:10.1007/s00787-025-02784-9)
Supplement: Supplementary file 1 — Supplementary Material 1 [file 787_2025_2784_MOESM1_ESM.docx]

**Table S1 Therapeutic alliance studies using one-with-many analyses: Variance partitioning, reciprocity and relation with treatment outcome**

|  |  | **Proportion of variance** | | | | **Reciprocity** | | **Association with treatment outcome** |
| --- | --- | --- | --- | --- | --- | --- | --- | --- |
| **Study** | **Rater** | **Perceiver** | **Partner** | **Client**  **relationship** | **Therapist**  **relationship** | **Generalised**  **reciprocity** | **Dyadic**  **reciprocity** |  |
| Marcus 2009 | Client | – | 6.4% | 93,6% * | – | r = -0.29 | r = 0.36 * | • Client relationship effect pos. related with outcome (p<0.05)  • Therapist partner effect neg. related with outcome (p<0.001) |
|  | Therapist | 30.0% * | – | – | 70.0% * |  |  |  |
| Marcus 2011 | Client | – | 5.4% | 94.6% * | – | r = 0.82 ** | r = 0.53 * | • Client relationship effect pos. related with outcome (p<0.003) |
|  | Therapist | 33.3% * | – | – | 66.7% * |  |  |  |
| Uckelstam 2020 | Client | – | 7.1% | 92.9% * | – | r = 0.52 * | r = 0.38 * | • Client relationship effect pos. related with outcome (p<0.01)  • Therapist relationship effect pos. related with outcome (p<0.01) |
|  | Therapist | 45.0%* | – | – | 55.0% * |  |  |  |
| van Benthem 2025 | Client | – | 0.8% | 99.2% ** | – | r = 0.01 | r = 0.14 | • Client relationship effect pos. related with outcome (p<0.001) |
|  | Therapist | 44.0%* | – | – | 56.0% ** |  |  |  |
| Hagiwara 2014 ^(a)^ | Client | – | 8.6% | 91.4% ** | – | r = 0.45 | r = 0.01 | • Therapist relationship effect pos. related with outcome (p<0.02) |
|  | Therapist | 43.3%* | – | – | 56.7% ** |  |  |  |

^(a)^ The study of Hagiwara et al. (2014) did not investigate therapeutic alliance, but instead focused on 'perceived teamness' among physicians and their patients

* p < 0.05

** p < 0.001
